# Epidemiology of sexually transmitted infections: trends among patients screened for sexually transmitted infections in rwandan health facilities 2014–2020

**DOI:** 10.1186/s12879-022-07685-9

**Published:** 2022-08-20

**Authors:** Jean Damascene Makuza, Phyumar Soe, Dahn Jeong, Marie Paul Nisingizwe, Donatha Dushimiyimana, Justine Umutesi, Ladislas Nshimiyimana, Clarisse Maliza, Janvier Serumondo, Eric Remera, Gallican Nshogoza Rwibasira, Albert Tuyishime, David J. Riedel

**Affiliations:** 1 School of Population and Public Health, University of British Columbia, 2613 Melfa Lane, Vancouver, BC Canada; 2 Clinical Prevention Services, British Columbia Centre for Disease Control, Vancouver, BC Canada; 3 Rwanda Biomedical Centre, Kigali, Rwanda; 4 Hannover Medical School, Hannover, Germany; 5 Institute of Human Virology, University of Maryland School of Medicine, Baltimore, MD USA

**Keywords:** Trends, Epidemiology, STIs, Syndromes, Rwanda

## Abstract

**Background:**

Sexually Transmitted Infections (STIs) are of great global health concern. Currently, there are limited epidemiological data characterizing STIs in the general population in Rwanda. We assessed the national and regional epidemiology of STIs in Rwanda from 2014–2020 among patients syndromically screened for STIs in all health facilities in Rwanda.

**Methods:**

This is a retrospective analysis of the trend of STIs epidemiology among screened patients at all health facilities in Rwanda using data from the Health Management Information System (HMIS) reporting. Adult patients (15 years and over) screened for STIs between July 2014 and June 2020 were included in the analysis. Outcomes of interest were the number of individuals screened for STIs and individuals diagnosed with at least one STI with a syndromic approach only or plus a test together.

**Results:**

Overall, the number of individuals screened for STIs over the study period was 5.3 million (M) in 2014–2015, 6.6 M in 2015–2016, 6.3 M in 2016–2017, 6.7 M in 2017–2018, 6.2 M in 2018–2019, and 4.9 M in 2019–2020. There was a modest increase in the number of individuals diagnosed and treated for STIs from 139,357 in 2014–15 to 202,294 (45% increase) in 2019–2020. At the national level, the prevalence of STI syndromes amongst individuals screened at health facilities in Rwanda varied between 2.37% to 4.16% during the study period. Among the provinces, Kigali city had the highest prevalence for the whole 6 years ranging from 3.46% (95%CI: 3.41, 3.51) in 2014–2015 to 8.23% (95%CI: 8.15, 8.31) in 2019–2020.

**Conclusion:**

From 2014 to 2020, the number of patients screened for STI syndromes in Rwanda varied between 4.9 M and 6.7 M. However, the prevalence of STIs among screened patients increased considerably over time, which could be associated with public awareness and improved data recording. The highest prevalence of all STIs was observed in urban areas and near borders, and private clinics reported more cases, suggesting the need to improve awareness in these settings and increase confidentiality and trust in public health clinics.

## Background

Sexually transmitted infections (STIs) are an important global health concern as more than 1 million people are newly infected with the four most curable STIs each day [1]: *Treponema pallidum*, *Neisseria gonorrhoeae*, *Trichomonas vaginalis,* and *Chlamydia trachomatis* [2, 3]. This burden has been disproportionally high among low and middle income countries, and African countries account 20% of all STIs with an estimated prevalence of 12% for trichomoniasis, 4% for gonorrhea, 2% for chlamydia, and 1.5% for syphilis [4] in the general population.

Despite sensitization for behavior change to key populations for STIs and HIV, the number of STIs is still increasing in different African countries. Therefore, other measures such as systematic screening, testing, and treatment for most at-risk populations and their partners are still needed for better management of this burden [5]. A meta-analysis published in 2018, has shown that in Southern/East African regions, among women, higher-risk populations such as sex workers had a higher prevalence of gonorrhea and syphilis than clinic/community-based populations [6].

Factors associated with the acquisition of STIs include lack of STI prevention information, having multiple sexual partners, and inconsistent use of condoms [7–9]. As some STIs are asymptomatic [10], STI prevalence is usually underestimated, and many patients seek treatment at late stages of infection, which may contribute to irreversible complications. Late diagnosis is not only due to asymptomatic STIs but also due to stigmatization at health facilities, and so many patients self-medicate at home [11].

Timely diagnosis and treatment of STIs are often hampered by the lack of symptoms, inadequate and/or poor availability of diagnostics, and inaccessibility and low quality of treatment in resource-limited settings [12]. As data on STI burden are critical to guide STI prevention and control activities, the first strategic direction of the Global Strategy for STIs prevention and control is to increase information, including STIs prevalence estimates, for focused public health action [13].

The 2019–2020 Rwanda Demographic Health Survey (RDHS) estimated that about 4.4% of the general population between 15–49 years old reported having at least one STI symptom during the last 12 months before the survey[14]. A nationwide household-based survey done in Rwanda in 2013 found a prevalence of syphilis in the general population of 0.9% with a higher prevalence in 25–49 year olds compared to those between 15–24 years old, women who only attained primary school or no-education compared to those who attended secondary/vocational/high education, and people living with HIV in comparison to those without [15].

In 2010, the Rwandan Ministry of Health (MOH) adopted the World Health Organization (WHO) STI guidelines for addressing the STI burden in Rwanda. These guidelines include a systematic screening for STIs at every health facility, which consists of assessing STI signs and symptoms for everyone who consults at the health facility; if a patient is diagnosed with an STI, syndromic management and treatment of STIs is provided at the health center or hospital in lieu of appropriate diagnostic materials. In addition to providing the treatment for individuals, partners of diagnosed received presumptive treatment. Capacity building of healthcare workers continues to play an important role in STIs management and its prevention and control in Rwanda.

WHO guidelines and United States Center for Diseases Control (US CDC) recommend the syndromic management of STIs using specific algorithms and treatment options for the major STI signs and symptoms: genital ulcers, penile discharge, abdominal pain, vaginal discharge (i.e., leucorrhea) in settings where resources are limited [16, 17]. The use of a syndromic approach has been linked with many limitations such as considering only symptomatic cases, over-treating pathogens which may not be present, or considering some syndromes as STIs while they are not, e.g., bacterial vaginosis. A study conducted in Rwanda in 2016 using a targeted point-of-care testing compared with syndromic management of urogenital infections in women showed that the majority of women with vaginal discharge had bacterial vaginosis and Trichomonas vaginalis. This study suggest the utility of targeted point-of-care testing over a syndromic approach [18]. However, the cost of those targeted point-of-care testing is still high in resources-limited countries like Rwanda and make syndromic approach the most appropriate in the Rwandan context.

According to the 2019 Rwanda Guidelines for management of STIs and viral hepatitis, syndromic approach is recommended in management of STIs at health center which is the second lowest primary health facility in Rwandan health system and etiologic approach at hospitals and in other health facilities which have means for STIs testing [19]. Since 2012 in Rwanda, all patients consulting health facilities for any pathology are supposed to be screened for STIs and diagnosed and treated if found to have an STI. Individuals treated for STIs are recommended to visit the health facility one week after treatment for the follow up visit for checking of treatment effect and adherence to treatment and STIs prevention methods[19]..

Most studies conducted on STIs in Rwanda are confined to specific populations such as female sex workers [10]**,** MSM [20, 21]**,** and people living with HIV [22]**,** and even those surveys are self-reported and missed people diagnosed at health facilities. Additionally, data trends for consecutive years among the general population is limited. Hence, there is a need to explore the trends of epidemiology of STIs in the general population. As from 2014 when indicators on STIs were updated, there was no study exploring the trends of STIs profile in Rwanda; this study aimed to assess the epidemiological trends of STIs and their distributions in different Rwandan regions from 2014–2020 among the general population screened for STIs in all health facilities.

## Methods

### Study design

The study was a secondary data analysis assessing the trend of STI epidemiology among screened patients at all health facilities in Rwanda from July 2014 to June 2020 using data from the HMIS reporting system. HMIS is a monthly reporting system comprising data from all health facilities in the country. HMIS is a web-based open-source platform (District Health Information System 2 [DHIS2]) and has been in Rwanda since January 2012 to collect routine data for healthcare service utilization [23]. Since 2011, the Rwandan Ministry of Health has initiated routine/systematic STI screening for all people visiting health facilities (HFs), consisting of assessing STI signs and symptoms through interrogation and/or examination of all individuals consulting in each health facility. Indicators on STIs were updated in 2014, and diagnosis of STI is defined based on the 2015 Rwanda Guidelines, updated in 2019 [19]. The data collected from all health facilities included screening, diagnosis, and treatment data. Majority of data were shown to be of high quality according to study evaluating the quality of HMIS data conducted in 2020 where high proportions of health facilities achieved acceptable verification factors for data on different indicators in 4 districts [24].

We included all adult patients (15 years and older) who visited private and public health facilities for any health reason, and received a screening for vaginal discharge, urethral discharge, genital ulcers, syphilis, epididymitis, pelvic inflammatory diseases (PID), and other STI syndromes in all health facilities in all districts of Rwanda from July 2014 to June 2020. We excluded children below 15 years old and those screened before July 2014 and after June 2020.

### Data collection procedures

Aggregate number of people screened, diagnosed, and treated are electronically recorded in HMIS. Data was confidentially recorded in Microsoft Excel from the HMIS data collection form. Collected data included number of patients screened, diagnosed and treated according to the type of health facility, location (district and province), and year. Data was from the nationwide STIs screening, diagnosis, and treatment of 2014–2015 to 2019–2020 periods.

### Variables of the study

Outcomes of interest were the number of individuals screened for STIs and individuals who were diagnosed with at least one STI with syndromic approach only or plus a test for syphilis when applicable together. These include genital ulcers, genital discharge for women, urethral discharge for men, inguinal bubo, painful swelling of scrotum, pelvic pain in women or pelvic inflammatory disease, and genital warts or Condyloma accuminata. Additional variables were district and province where STIs services were delivered, type of health facility and period of screening. Rwanda is organized into 4 provinces which have 30 districts, one province (Kigali city) is totally composed of 3 urban districts while other provinces are composed of at least 1 or 2 semi-urban districts and several rural districts. The South province includes 2 semi-urban and 6 rural districts; the North includes 1 semi-urban and 4 rural districts, the East includes 2 semi-urban and 5 rural districts, and the West includes 2 semi-urban and 5 rural districts.

The Rwanda health system is organized into public and private health facilities. The public health facilities include 8 referral hospitals, 4 provincial hospitals affiliated to the 4 administrative provinces, 36 district hospitals affiliated with 30 administrative districts, and 510 health centers and prison clinics affiliated with 416 administrative sectors. Private health facilities include private hospitals, private clinics, private polyclinics and dispensaries[25].

### Statistical analysis of data

Prevalence was calculated for different provinces and types of health facilities in each year from 2014–2015 till 2019–2020. Prevalence of STIs and 95% confidence Interval (CI) were calculated using the total STIs or individual syndrome as numerator and the total number of patients screened as the denominator. R version 4.0.2 was used in the data cleaning and analysis. QGIS version 2.0.1 was used to draw maps of STIs prevalence by district for different years.

### Ethical clearance

Rwanda Biomedical Center Research committee has approved the conduct of this study. The extraction of data from the database was granted by Rwanda Biomedical Center (No. 3310/RBC/2021). The access and utilization of data was confidential and restricted to investigators involved in the analysis. The need for written informed consent was waived by the ethics committee/Institutional Review Board of Rwanda Biomedical Center, because of the retrospective nature of the study. I confirm that all experimental protocols were approved by Rwanda Biomedical Center.

## Results

### General characteristics of population screened and those diagnosed and treated for STIs

**Fig. 1:**
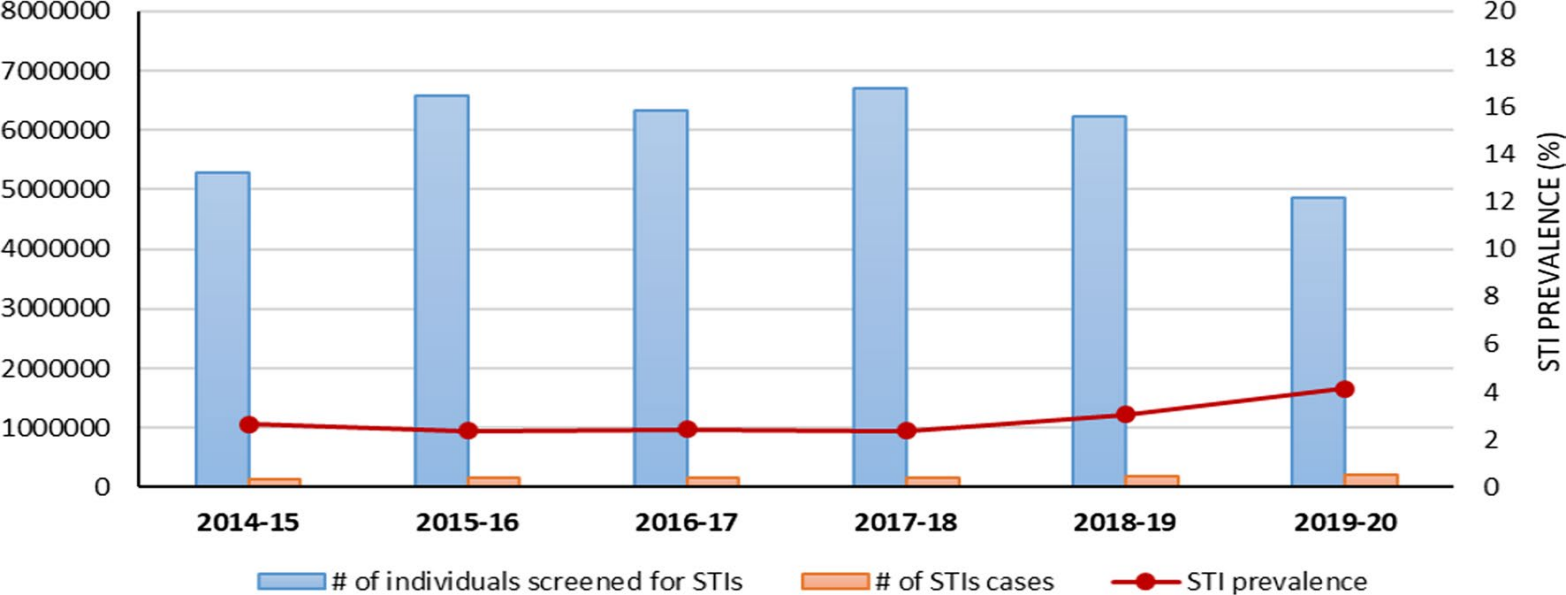
Trends of individuals screened, those diagnosed and treated for STIs and prevalence of STIs from 2014 to 2020

Overall, the number of individuals screened for STIs has increased over the years, from 5.3 million in 2014–2015 to 6.2 million 2018–2019. However, the total number of individuals screened for STIs declined abruptly in 2019–2020, reaching the lowest recorded ever during the study period (about 4.9 million). This figure represents more than 20% decrease compared to 2018–2019. The peak for screening was seen in 2017–2018, reaching 6.7 million individuals screened for STIs (Fig. 1 and Table 1). There has been a modest increase in the number of individuals diagnosed and treated with STIs over the study period, from 139,357 in 2014–2015 to 202,294 in 2019–2020.

**Table 1:** Number of patients Screened, diagnosed and treated for STIs by Province and type of health facility

	2014–15		2015–16		2016–17		2017–18		2018–19		2019–20	
	# of people screened N (%)	Diagnosed and treated for STI N (%)	# of people screened N (%)	Diagnosed and treated for STI N (%)	# of people screened N (%)	Diagnosed and treated for STI N (%)	# of people screened N (%)	Diagnosed and treated for STI N (%)	# of people screened N (%)	Diagnosed and treated for STI N (%)	# of people screened N (%)	Diagnosed and treated for STI N (%)
Province												
East	1,138,099 (21.53)	34,126 (24.49)	1,531,669 (23.26)	42,347 (27.17)	1,358,425 (21.43)	44,835 (28.97)	1,456,251 (21.77)	48,530 (30.51)	1,162,147 (18.67)	58,503 (30.83)	864,882 (17.79)	61,861 (30.58)
Kigali City	464,978 (8.80)	16,080 (11.54)	507,737 (7.71)	19,575 (12.56)	442,892 (6.99)	16,908 (10.93)	563,637 (8.43)	19,667 (12.36)	552,694 (8.88)	30,602 (16.13)	434,763 (8.94)	35,792 (17.69)
North	737,378 (16.95)	22,462 (16.12)	947,311 (14.38)	24,353 (15.63)	1,018,977 (16.08)	21,104 (13.64)	1,103,087 (16.49)	21,585 (13.57)	1,114,859 (17.91)	22,131 (11.66)	891,514 (18.33)	25,693 (12.70)
South	1,558,225 (29.48)	30,086 (21.59)	1,777,344 (26.99)	28,293 (18.16)	1,671,678 (26.38)	29,162 (18.84)	1,624,989 (24.29)	27,380 (17.21)	1,537,903 (24.71)	34,591 (18.23)	1,207,870 (24.84)	36,540 (18.06)
West	1,386,487 (26.23)	36,603 (26.27)	1,821,389 (27.66)	41,267 (26.48)	1,845,668 (29.12)	42,746 (27.62)	1,942,077 (29.03)	41,894 (26.34)	1,855,924 (29.82)	43,950 (23.16)	1,463,580 (30.10)	42,408 (20.96)
Total	5,285,367 (100)	139,357 (100)	6,585,450 (100)	155,835 (100)	6,337,640 (1000	154,755 (100)	6,690,041 (100)	159,056 (1000	6,223,527 (100)	189,777 (100)	4,862,609 (100)	202,294 (100)
*Total Patients who visited HFs (% of people screened for STIs)	NA		NA		17,328,989 (36.57)		18,878,021 (35.43)		19,026,514 (32.71)		18,860,619 (25.78)	
Type of facility												
District Hospital	457,403 (8.65)	7637 (5.48)	508,058 (7.71)	6694 (4.30)	441,747 (6.97)	6690 (4.32)	394,334 (5.89)	5966 (3.75)	371,914 (5.98)	6604 (3.48)	314,689 (6.47)	6795 (3.36)
Health Center	4,708,252 (89.08)	128,099 (91.92)	5,940,478 (90.21)	144,551 (92.76)	5,758,670 (90.86)	143,410 (92.67)	6,149,158 (91.92)	149,449 (93.96)	5,695,445 (91.51)	178,132 (93.86)	4,401,291 (90.51)	191,809 (94.82)
Prison clinics	51,141 (0.97)	408 (0.29)	57,160 (0.87)	665 (0.43)	54,117 (0.85)	788 (0.51)	55,712 (0.83)	765 (0.48)	64,073 (1.03)	999 (0.53)	66,386 (1.37)	1232 (0.61)
Private clinics	7264 (0.14)	1152 (0.83)	12,610 (0.19)	2450 (1.57)	15,041 (0.24)	2874 (1.86)	13,487 (0.20)	1847 (1.16)	16,768 (0.27)	3204 (1.69)	11,088 (0.23)	1755 (0.87)
Provincial Hospital	32,500 (0.61)	964 (0.69)	37,160 (0.56)	1161 (0.75)	34,679 (0.55)	680 (0.44)	39,093 (0.58)	701 (0.44)	28,179 (0.45)	374 (0.20)	30,563 (0.63)	321 (0.16)
Referral Hospital	28,607 (0.54)	1097 (0.79)	29,984 (0.46)	314 (0.20)	33,386 (0.53)	313 (0.20)	38,257 (0.57)	328 (0.21)	47,148 (0.76)	464 (0.24)	38,592 (0.79)	382 (0.19)

*HFs* Health facilities, *NA* Not available, *The total number of people who visited both public and private health facilities [26]

At the provincial level, the proportion of screened individuals was consistently higher in the Western (26.23% of all people screened in 2014–2015 to 30.10% 2019–2020) and Southern (29.48% of total people screened in 2014–2015 to 24.84% 2019–2020) provinces than other provinces over the years. The proportion of diagnosed STI cases were highest in the Eastern (24.49% of the total STI syndromes diagnosed in 2014–2015 to 30.58% 2019–2020) and Western (26.27% of the total STI syndromes diagnosed in 2014–2015 to 20.96% 2019–2020) provinces while the lower numbers of cases were seen in Kigali city and Northern province for the whole period (Table 1).

Over the study period, the vast majority of patients were screened, diagnosed and treated at health center level whereas District, Provincial and Referral hospital served fewer individuals for screening and STI treatment services. See more details in Table 1.

### STI syndrome prevalence by province and type of facility

**Fig. 2:**
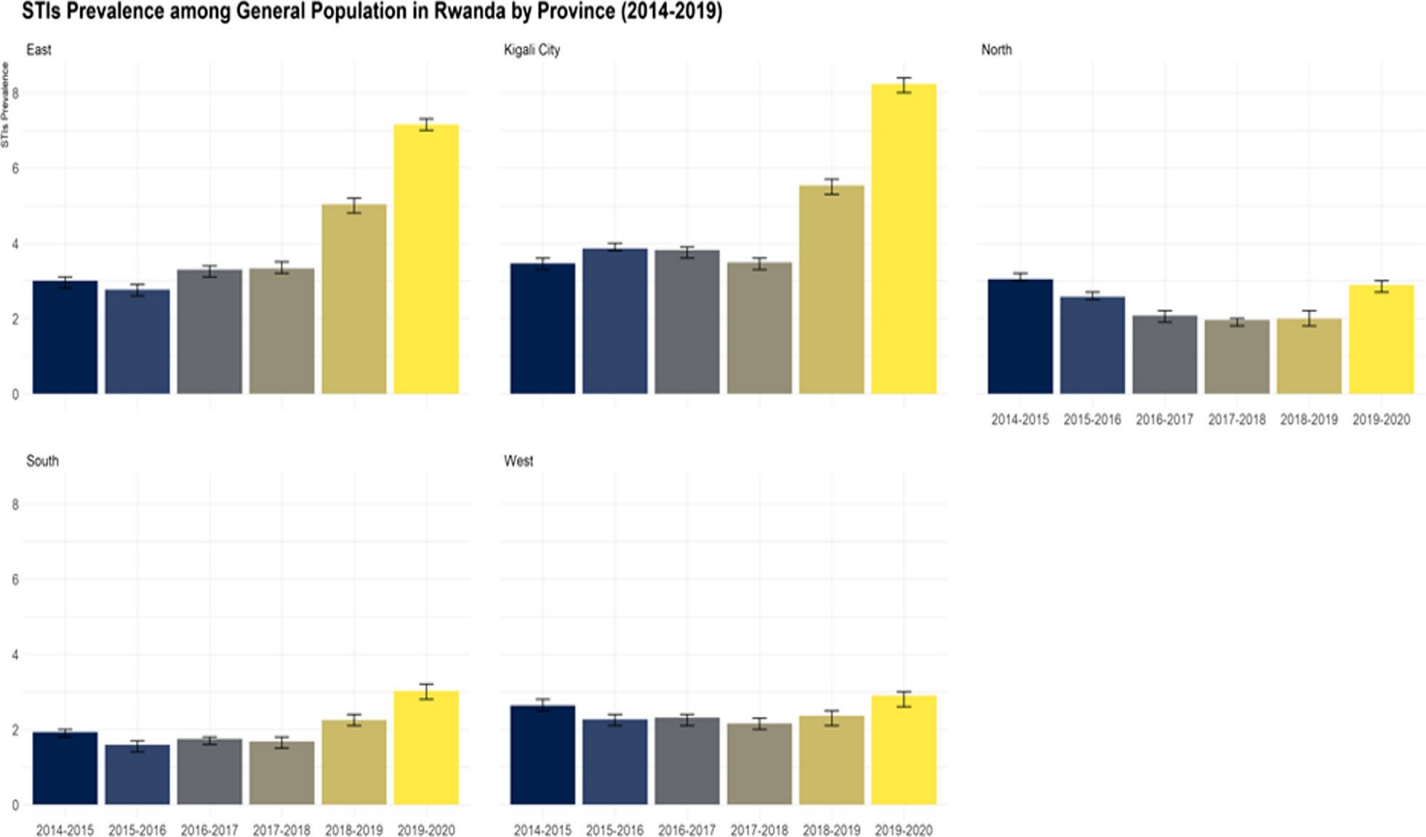
Trends of STIs prevalence among patients attending health facilities in Rwanda and by province (2014–2020)

At the national level, there has been a steady increase of STI syndrome prevalence amongst heath facility attendees over the years, and the trend reached its peak in 2019–2020 at 4.16% (95% CI 4.14, 4.18). Among the provinces, Kigali city had the highest prevalence for the whole 6 years ranging from 3.46% (95%CI: 3.41, 3.51) in 2014–2015 to 8.23% (95%CI: 8.15, 8.31) in 2019–2020. Southern province had the lowest prevalence from 2014–2015 to 2018–2019 with variation from 1.59% (95%CI: 1.57, 1.61) in 2015–2016 and 2.25% (95%CI: 2.22, 2.27) in 2018–2019. However, in 2019–2020, the Northern province was the province with the lowest prevalence at 2.88% (95%CI: 2.85, 2.92). See more details in Fig. 2 and Table 2.

**Table 2:** STI syndrome prevalence by Province and Type of Facilities

	2014–15		2015–16		2016–17		2017–18		2018–19		2019–20	
	%	95% CI	%	95% CI	%	95% CI	%	95% CI	%	95% CI	%	95% CI
Province												
East	3.00	2.97, 3.03	2.76	2.74, 2.79	3.30	3.27, 3.33	3.33	3.30, 3.36	5.03	4.99, 5.07	7.15	7.10, 7.21
Kigali City	3.46	3.41, 3.51	3.86	3.80, 3.91	3.82	3.76, 3.87	3.49	3.44, 3.54	5.54	5.48, 5.60	8.23	8.15, 8.31
North	3.05	3.01, 3.09	2.57	2.54, 2.60	2.07	2.04, 2.10	1.96	1.93, 1.98	1.99	1.96, 2.01	2.88	2.85, 2.92
South	1.93	1.91, 1.95	1.59	1.57, 1.61	1.74	1.72, 1.76	1.68	1.66, 1.70	2.25	2.22, 2.27	3.03	2.99, 3.06
West	2.64	2.61, 2.67	2.27	2.24, 2.29	2.32	2.29, 2.34	2.16	2.14, 2.18	2.37	2.34, 2.39	2.90	2.87, 2.92
Overall prevalence	2.64	2.61, 2.65	2.37	2.34, 2.38	2.44	2.42, 2.45	2.38	2.36, 2.39	3.05	3.03, 3.06	4.16	4.14, 4.18
Type of facility												
District Hospital	1.67	1.63, 1.71	1.32	1.29, 1.35	1.51	1.48, 1.55	1.51	1.48, 1.56	1.78	1.73,1.82	2.16	2.11,2.21
Health Center	2.72	2.70, 2.74	2.43	2.42, 2.45	2.49	2.48, 2.50	2.43	2.42, 2.44	3.12	3.11, 3.14	4.36	4.34, 4.38
Prison clinics	0.80	0.72, 0.88	1.16	1.08, 1.25	1.46	1.36, 1.56	1.37	1.28, 1.47	1.56	1.46, 1.66	1.86	1.75,1.96
Private clinics	15.90	15.0, 16.72	19.4	18.74, 20.13	19.11	18.48,19.75	13.69	13.12,14.28	19.12	18.52,19.71	15.83	15.15,16.52
Provincial Hospital	2.97	2.78, 3.15	3.12	2.95, 3.31	1.96	1.82, 2.11	1.79	1.67, 1.93	1.33	1.20,1.47	1.05	0.94,1.17
Referral Hospital	3.83	3.62, 4.06	1.05	0.94, 1.17	0.94	0.84, 1.05	0.86	0.77, 0.95	0.98	0.90, 1.08	0.99	0.89,1.09

In terms of type of health facility, the highest STI prevalence was found at private clinics for the whole study period with the highest prevalence of 19.4% (95%CI: 18.74, 20.13) in 2015–2016. The percentage of STI syndrome positive results was lowest at referral hospital level for the whole period except in 2014–2015. STI prevalence at prison clinics was the second lowest throughout the period, followed by STI prevalence in province hospitals. See more details in Table 2.

### Distribution of STIs according per district

**Fig. 3:**
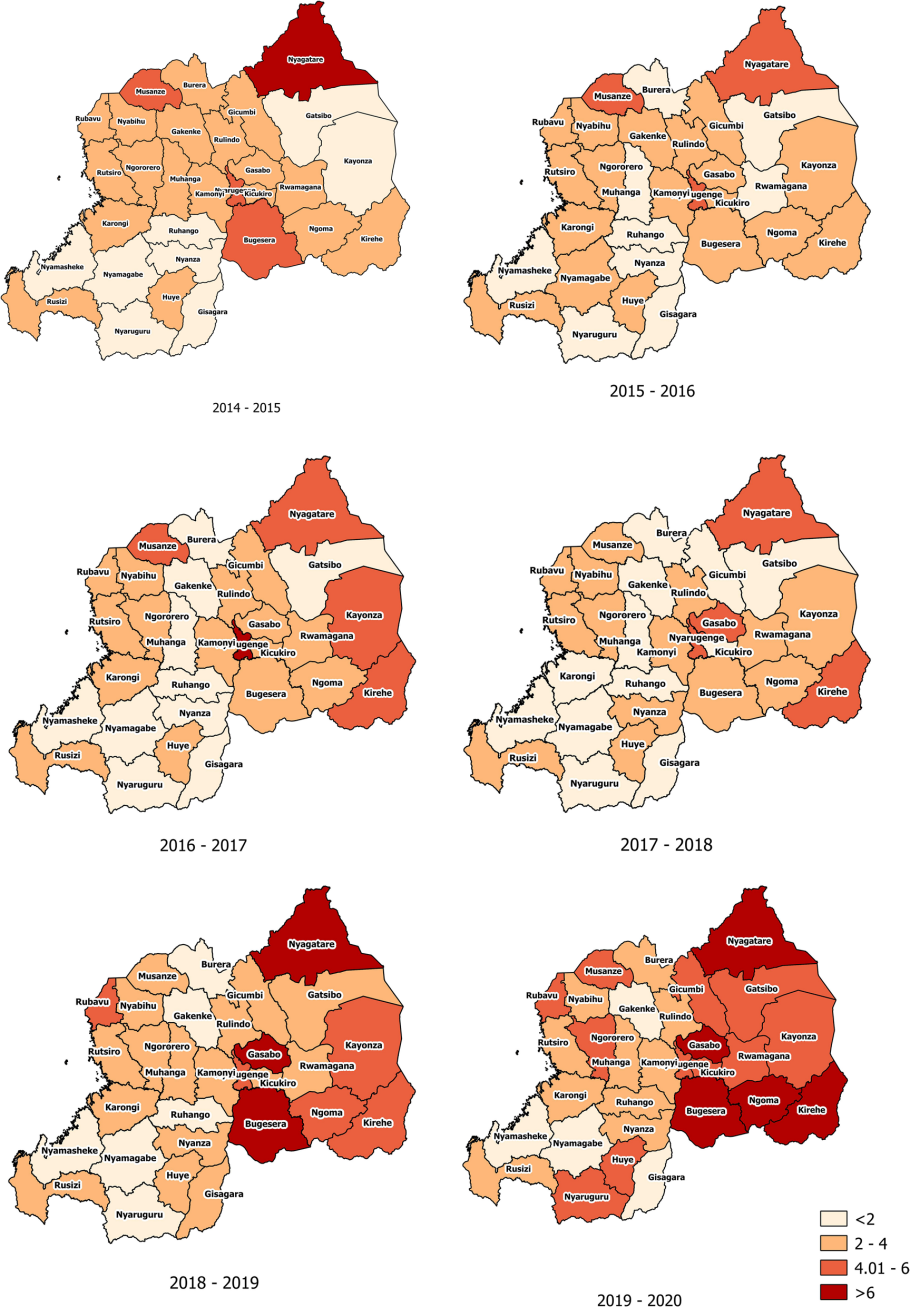
Distribution of diagnosed and treated STIs patients in 2014–2020

The STI prevalence was highest in Nyagatare district in Eastern province during the study period varying between 4 to 6% except in 2016–2017 when Nyarugenge had the highest prevalence with over 6%. During the period of 2015–2016, Nyagatare District in Eastern province, Musanze in Northern province, and Nyarugenge district in Kigali City were leading other districts in having the highest prevalence of STIs which were between 4.01–6%. During the period of 2016–2017, the highest prevalence was in Nyarugenge district in Kigali City with more than 6%. During the period of 2017–2018, the highest prevalence was in Nyarugenge and Gasabo districts in Kigali City, Nyagatare, and Kirehe districts in Eastern province with the prevalence of STIs between 4.01–6%. During the period of 2018–2019, the highest prevalence was found in Nyagatare and Bugesera Districts in Eastern province, and Gasabo district in Kigali city with more than 6%. In the period of 2019–2020, Nyagatare, Ngoma, Bugesera and Kirehe Districts in Eastern province, Musanze District in North and Gasabo district in Kigali City had the highest prevalence with more than 6%. Most of these districts with high prevalence are closer to neighboring countries. The remaining districts have the prevalence under 3.58%. See details in maps on Fig. 3.

**Fig. 4:**
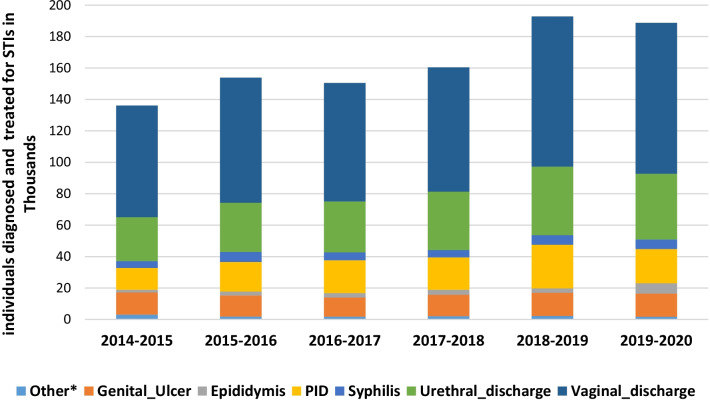
STIs syndromes from 2014–2015 to 2019–2020. *Other STI syndromes include genital warts, inguinal bubo, and painful swelling of scrotum

The most commonly reported STI syndrome was vaginal discharge over the study period, with the highest number presentation in 2019–2020. Male urethral discharge was the second most common symptom over the study period. See more details in Fig. 4.

## Discussion

This study aimed at assessing the nationwide population-based epidemiology of STIs among general population patients screened at different health facilities in Rwanda from 2014 to 2020. The total number of patients screened and the number of cases of documented STIs among screened patients increased over the period, however, the percentage of people screened among those who visited the health facilities from 2016 to 2020 decreased. This study showed the highest prevalence of STIs was in Kigali City and Eastern Province. Although the majority of patients are screened, diagnosed, and treated at health center level, the highest prevalence of STIs was seen in private clinics. This study also showed that some districts from Kigali City and majority of districts, which are closer to the borders with other countries especially in Eastern Provinces, were more likely to have higher STI prevalence over the period. The most prevalent STI syndrome reported during the study period was vaginal discharge.

Considering the number of patients screened, and those diagnosed and treated for STIs during the study period (from 2014 to 2020), the number of patients screened in 2014–2015 are two times higher than those screened one previous years (2013–2014). They are 10 times higher than that of the period of 2010–2011 for the patients screened while the number of STIs diagnosed and treated increased from 2 to 5 times when compared to the period of 2010–2011 and 2013–2014 [27]. Data from 2010–2011 were reported before implementation of different strategies for prevention and Control of STIs adopted by Rwanda Ministry of Health. Before 2013–2014, methodology for screening of STIs, and strategies for STIs reporting changed and were different from that is being used from 2013–2014 where only people with complains related to STIs were considered for STIs screening and majority of data were not reported.

This increasing coverage of screened, diagnosed and treated patients for STIs in Rwanda is probably the results of increased training of health care providers from all health facility level, provided as implementation of Rwanda HIV National Strategic Plan 2013–2018 established strategies aimed at increasing systematic STI screening and treatment in all patients who consult health facilities [28]. Other potential contributors to this success include the establishment of a unit in charge of STIs in the Rwanda Biomedical Centre, and enhanced monitoring and evaluation for STIs and HIV. However, according to available data in the MOH, the percentage of people screened for STIs by total population who visited the health facilities decreased over the years from 36.57% in 2016–2017 to 25.78% in 2019–2020 [26]. There is a need of further strengthening the STI program by training of more health care providers especially at health center level where most STIs cases are diagnosed and treated. Strengthening monitoring and evaluation is also needed for increasing the rate of syndromic screening for health facility attendees.

In this study, the overall prevalence of STIs among patients screened for STIs was 2.6% in 2014–2015, 2.4% in 2015–2016, 2016–2017, and in 2017–2018, 3.0% in 2018–2019 and 4.1% in 2019–2020. The highest prevalence in 2019–2020 could be due to the impact of the COVID-19 pandemic [29] where on one way the number of patients screened was reduced while the number of patients diagnosed and treated for STIs increased because majority of cases who consulted were likely presenting with acute STI symptoms while for another way health care providers did not perform STIs screening for a big number of population who visited the HFs. Individuals with more pronounced symptoms and presentations may have been more likely to visit a healthcare facility compared to previous years.

The most common STI syndrome in this study was vaginal discharge syndrome. Predominance of abnormal vaginal discharge was reported also in a cross-sectional study conducted among HIV-negative sex workers in Rwanda [12]. RDHS 2020 has reported a prevalence of 4.4% of STIs among women with the predominance of abnormal genital discharge and genital ulcer (13.3%)[14]. The predominance of vaginal discharge as an STI syndrome was also reported in the study exploring the prevalence of STIs using a syndromic approach in India that has found the predominance of vaginal discharge syndrome with 51.7% [30]. In addition to reduced rate of syndromic screening for health facility attendees, the majority of STIs are still likely to be missed due to their asymptomatic nature, given the increasing body of evidence demonstrating the limitations of syndromic screening and management. The government should look how to establish targeted point-of –care testing but in the meantime, the rate of people screened for STIs could be increased.

In this study, the prevalence of STIs is very high for patients screened at private clinics, ranging between 15.69% and 19.4% for the whole study period. The reason behind this high prevalence could be the stigma and discrimination experienced by patients suffering from these infections. It is possible that people who felt stigmatized chose to visit private clinics where they will meet fewer people instead of local or public health centers. Rwanda DHS has reported experience of stigma as 37% among men and 50% among women living with HIV or other STIs [25]. Literature revealed that in Africa STI-related stigma was significantly associated with a decreased odds of STI testing and a decreased willingness to notify partners of an STI which sometimes leads to a preference for traditional healers who are still viewed by many as having the best treatment for STIs even though the efficacy of such treatments remains unproved [31]. Therefore, there is a need to reduce the stigma related to STIs by providing integrated services, health education, wider options to STIs management, aid disclosure and partner notification practices.

Findings from this study showed that there are districts which are more affected by STIs than others. These districts are known to be home to more at risk people for STIs like female sex workers and truck drivers due to availability of means that meet their daily needs. It has been shown that sex workers and truck drivers are more engaged in high-risk sexual behaviors such as multiple sexual partnerships, and low consistent condom use which increase the risk of getting and transmit STIs [15, 32, 33]. Other reasons for this high prevalence among these districts include the high mobility from different countries including those with high prevalence of HIV and STIs, non-adherence to programs aimed at HIV/AIDs and STIs prevention like consistent condom use, and PrEP. More efforts need to be put in STIs prevention and management for most at risk populations living mainly in Kigali City and in some districts closer to borders with other countries. A recent publication on key populations in Kigali found that prevention and timely treatment in key populations including FSWs are lacking which highlight the need of establishment of HIV and STIs prevention programs like tailored and integrated HIV/STIs programs [34].

This study has several strengths including the nationwide sample size and representative of all health facilities countrywide were considered which increase the external validity as well as the estimates over time of STIs in Rwanda. The STI prevalence from this study are comparable with other studies like DHS- 2015 and DHS 2020 [14, 25]. To date in Rwanda few studies interpret district-level data, as this study did it, this is an added value to results interpretation.

However, different limitations were noted. Firstly, there are limited variables due to aggregate data, having more variables like sex and age group could explain more the cause of change or no change of STIs epidemiology in one or another region. Secondly, lack of individualized data could lead to ecological fallacy, so there a need to conduct another study with individualized data for confirming the STIs distribution according to different factors. Thirdly, on one hand most STIs were diagnosed based on syndromes, then the exact cause of the STI syndrome remains unknown. This means that people could have multiple STIs when presenting with a specific syndrome. For example, vaginal or urethral discharge could have Chlamydia and Gonorrhea and even be co-infected with syphilis, HSV, or something else at the same time. On the other hand, there is a possibility of over-diagnosis for vaginal discharge, given that bacterial vaginosis is not an STI, and is a very common cause of vaginal discharge. We cannot forget that there are several cases of asymptomatic STIs which may underestimate the number of reported STIs. Lastly, there is a problem of data accuracy due to its nature, so there is a possibility that some data were not well collected or there is under- or over-estimation of STI prevalence. These are only diagnosed cases at health facilities. People who go to seek care might not be representative of the general population. Future studies on patient-level are recommended for assessing the factors associated to STIs among Rwandan population.

To conclude, this study showed that from 2014 to 2020, in Rwanda, the number of individuals screened for STIs did not vary significantly, but the reported cases of STIs as well as its prevalence among screened patients increased. This study reports the highest prevalence of total STIs in urban area of Rwanda and in regions bordering other countries. The majority of patients are screened, diagnosed and treated at the peripheral level; however, the high prevalence of STIs was seen in the private clinics. There is a necessity to strengthen STIs prevention measures in the general population, with increased efforts to reach most at risk population. More trainings for health care workers and providing screening and testing materials and effective treatment at health centers and even in private clinics are encouraged. Results from this study will provide important knowledge about the impact of different measures taken by the Rwanda Ministry of Health and inform strategic direction and program planning.

## Data Availability

The study data, and data collection instruments are available upon request from the corresponding author. Forms and data can be accessed by written request to the corresponding author. The data will be made available following evaluation and approval of proposed use by the study.

## Abbreviations

DHIS2: District Health Information System 2
HFs: Health facilities
HIV: Human Immuno-deficiency Virus
HMIS: Health Management Information System
MSM: Men who have sex with men
MOH: Ministry of Health
PID: Pelvic inflammatory diseases
RDHS: Rwanda Demographic Health Survey
STIs: Sexually transmitted infections
US CDC: United States Center for Diseases Control
WHO: World Health Organization
